# Functional analysis of B and C class floral organ genes in spinach demonstrates their role in sexual dimorphism

**DOI:** 10.1186/1471-2229-10-46

**Published:** 2010-03-12

**Authors:** D Noah Sather, Maja Jovanovic, Edward M Golenberg

**Affiliations:** 1 Department of Biological Sciences, Wayne State University, Detroit, MI 48202, USA; 2 Current address: Seattle Biomedical Research Institute, 307 Westlake Avenue N, Seattle, WA 98109, USA

## Abstract

**Background:**

Evolution of unisexual flowers entails one of the most extreme changes in plant development. Cultivated spinach, *Spinacia oleracea *L., is uniquely suited for the study of unisexual flower development as it is dioecious and it achieves unisexually by the absence of organ development, rather than by organ abortion or suppression. Male staminate flowers lack fourth whorl primordia and female pistillate flowers lack third whorl primordia. Based on theoretical considerations, early inflorescence or floral organ identity genes would likely be directly involved in sex-determination in those species in which organ initiation rather than organ maturation is regulated. In this study, we tested the hypothesis that sexual dimorphism occurs through the regulation of B class floral organ gene expression by experimentally knocking down gene expression by viral induced gene silencing.

**Results:**

Suppression of B class genes in spinach resulted in the expected homeotic transformation of stamens into carpels but also affected the number of perianth parts and the presence of fourth whorl. Phenotypically normal female flowers developed on *SpPI*-silenced male plants. Suppression of the spinach C class floral organ identity gene, *SpAG*, resulted in loss of reproductive organ identity, and indeterminate flowers, but did not result in additional sex-specific characteristics or structures. Analysis of the genomic sequences of both *SpAP3 *and *SpPI *did not reveal any allelic differences between males and females.

**Conclusion:**

Sexual dimorphism in spinach is not the result of homeotic transformation of established organs, but rather is the result of differential initiation and development of the third and fourth whorl primordia. *SpAG *is inferred to have organ identity and meristem termination functions similar to other angiosperm C class genes. In contrast, while *SpPI *and *SpAP3 *resemble other angiosperms in their essential functions in establishing stamen identity, they also appear to have an additional function in regulating organ number and identity outside of the third whorl. We present a model for the evolution of dioecy in spinach based on the regulation of B class expression.

## Background

The ABC model for floral development has been extensively tested and applied to a wide variety of angiosperm species and has been found to be broadly conserved on sequence, expression, and functional levels. Nonetheless, those few exceptions to the canonical *Arabidopsis/Antirrhinum *model have been illuminating in understanding the processes involved in the evolution of the present array of floral morphologies [1]. For example, expanded B class expression appears to be common in the Liliaceae and can explain the morphological similarities of first and second whorl organs [2-5]. A number of species in the basal dicots display an analogously modified B class expression domain consistent with a gradient in sterile and reproductive organ morphology [6]. Similarly, novel temporal and spatial expression domains have been associated with novel organ morphologies [7-9]. In contrast, the assumption of the general conservation of expression has been used to assign homology of highly derived organs to putative ancestral forms [10-14]. Lastly, evolution of the coding sequences and their regulation following gene duplications have lead to lineage specific partitioning of function or development of new gene functions [15,16].

Among the most extreme examples of evolution of the reproductive organs in flowers is the development of unisexual flowers. Unisexuality has evolved independently in all orders of angiosperms. Morphologically, there appears to be two ways in which unisexual flowers arise, one in which initiated organs are aborted during development, and one in which stamens and/or carpels are never initiated [17]. Golenberg and Freeman [18] argued that floral organ identity genes, particularly B and C class genes, will not be instrumental in the sex-determination regulatory process in those species that achieve unisexuality by organ abortion. In those species, altered temporal or spatial expression of these genes will likely be a secondary outcome of the degeneration of the organs. Several well studied species develop this way, including *Zea mays *[19], *Rumex acetosa *[20], *Cucumis sativus *[21], and *Silene latifolia *[22-24]. In comparison, B and C class genes are more likely to be directly involved in sex-determination in those species in which organ initiation is regulated. Studies in *Thalictrum dioicum *[25] and *Spinacia oleracea *[26,27] demonstrate that some of the B and C class paralogues are alternatively expressed in either male or female flowers.

Diploid cultivated spinach, *Spinacia oleracea*, develops by differential organ initiation [27,28]. Female, or pistillate flowers develop two sepaloid perianth organs in the first whorl, no organs in what would be the second and third whorls, and a single ovule and ovary in the fourth whorl. In contrast, male, or staminate flowers develop two sequential pairs of sepaloid organs in the first whorl, no petals in the second whorl, four stamens in the third whorl, and no organs in the fourth whorl. We have shown that both B and C class genes are either alternatively expressed or spatially regulated in a sex-specific manner. The spinach C class gene *SpAGAMOUS *(*SpAG*) is expressed early throughout the floral primordium before the emergence of floral organ primordia in both males and female [27]. Later in development, *SpAG *expression is sex-specific and becomes restricted to the microsporangial cells in males and the nucellus in females. In contrast, the spinach B class genes *SpPISTILLATA *(*SpPI*) and *SpAPETALA3 *(*SpAP3*) were found to have highly sex-specific expression patterns [26]. *SpAP3 *was found by RT-PCR and northern blot to be strongly expressed in male flowers and weakly expressed in female flowers, although expression was undetectable in female flowers by *in-situ *hybridization. *SpPI *was found to be expressed early in male floral development and not in female floral development at any stage. Given that spinach B class genes are expressed before the initiation of floral organ primordia in a sex-specific manner, we hypothesized that one or both are directly involved in sex determination in this species, with *SpPI *being the most likely agent. In contrast, early C class expression in both males and females would suggest that the later sex-specific spatial expression patterns are likely a consequence of a sex-specific regulatory program and are involved in the sexual dimorphism rather than in the sex determination, itself.

To test this hypothesis, we analyzed the function of the spinach B (*SpPI *and *SpAP3*) and C (*SpAG*) class genes by suppressing individual gene expression during floral development. We demonstrate that both B and C class genes retain organ identity function as first described in *Arabidopsis*. *SpAG *also functions both in microsporangial development in males and in meristem determination in females. The spinach B class genes *SpPI *and *SpAP3 *are also required for normal organ number, whorl development, and sex determination. The lack of detected allelic differences in B class genes in males versus females implies that gender-specific development is controlled through trans-acting regulators of B class expression. These results indicate that regulation of B class genes is a major control point in sex-determination in spinach.

## Results

### Infection with *pWSRi:SpAP3 *causes homeotic transformations in males and hermaphroditic flowers

**Figure 1:**
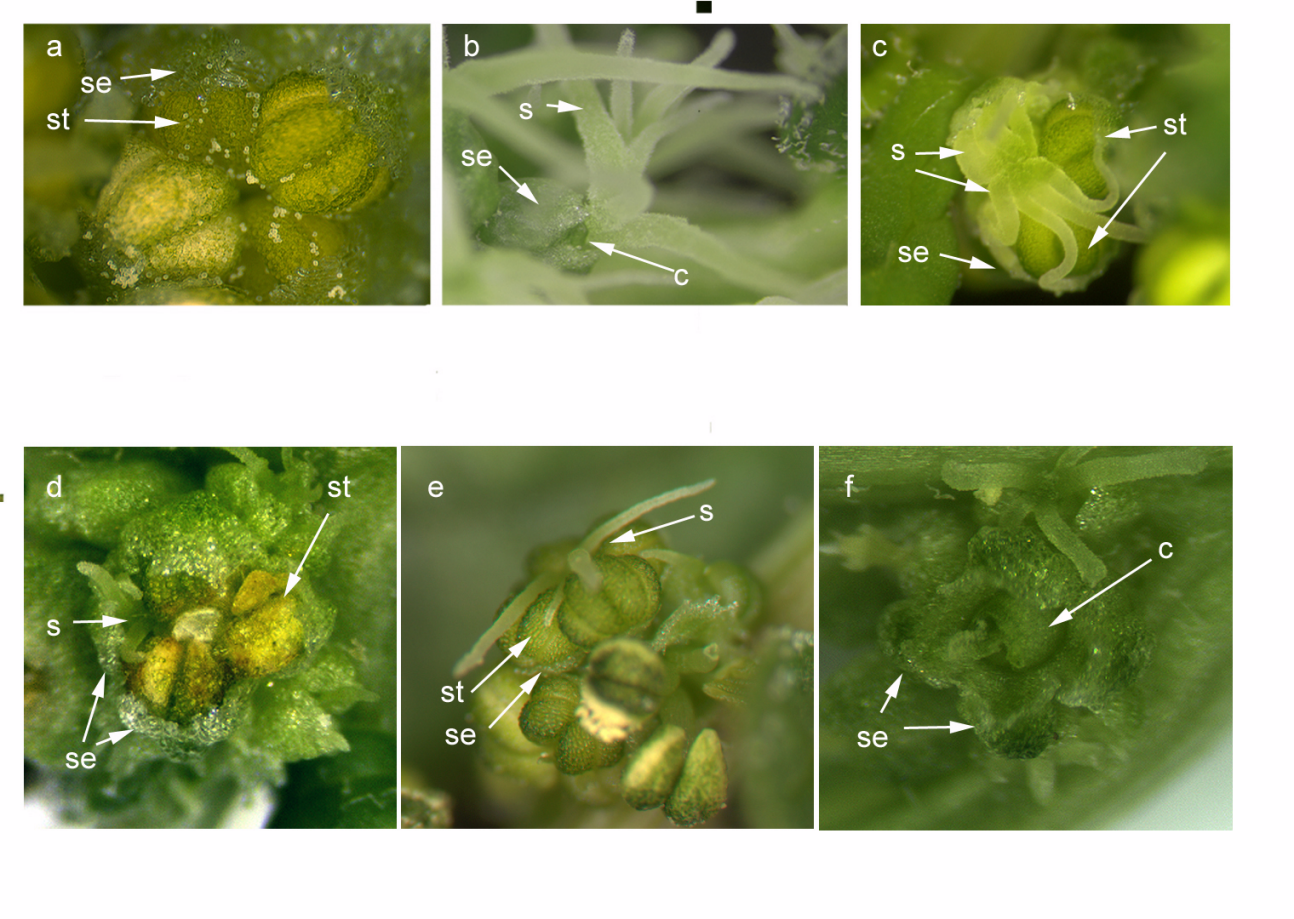
**SpAP3 silenced flowers**. Wild type spinach male flower (a) has four stamen (one designated with arrow) and four sepals (one designated with arrow). Wild type female flowers (b) with two sepals (one marked) that envelop the central carpel (marked) which develops a single ovuled ovary with usually four stigmatic arms. Flowers from *pWSRi:SpAP3 *treated plants (c through f). c and d. Stigmas from the developing carpels (arrows) are visible in the third whorl, along with stamens (arrows). In flower shown in c, there are an unusual six stigmatic arms. e. A hermaphroditic flower with a carpel developing in the fourth whorl, surrounding by four stamens and four sepals. f. A flower with a central (fourth whorl) carpeloid organ surrounded by four sepals. Abbreviations: st, stamens; se, sepals; c, carpel; s, stigma.

Spinach plants were treated with the gene silencing plasmid *pWSRi:SpAP3 *by biolistic bombardment with coated tungsten particles. Approximately five to six weeks post inoculation, all plants had transitioned to flowering. Approximately half of the original plants had differentiated into female plants. Wild-type female plants develop flowers with two sepaloid organs and a single central carpel (Figure 1b). The *pWSRi:SpAP3 *female plants were normal in appearance, with flowers developing two sepals and a single carpel. The female flowers were fertile, producing seeds after pollination. The remaining half of the treated plants differentiated into male. Wild-type male flowers develop four stamens and four sepaloid organs, with no central carpel (Figure 1a). All *pWSRi:SpAP3 *treated males had phenotypic defects in development in some flowers. Several flowers had homeotic transformations of stamens into carpels, producing flowers with mixed organs in the third whorl (Figure 1c and 1d). These mixed organ flowers did not develop a fourth whorl, but developed carpels in the third whorl in the place of stamens. Some carpels developed with more than the usual four stigmatic arms such as shown in Figure 1c. The stamens in the mixed organ flowers sometimes did not fully mature and produce pollen. A number of plants developed flowers that appeared to be fully hermaphroditic (Figure 1e) with four sepals, four stamens, and a single fourth whorl ovary. Some flowers had the normal complement of four sepals, but developed a single central carpel and no stamens (Figure 1f).

### Infection with *pWSRi:SpPI *causes homeotic transformations and floral gender transformation

**Figure 2:**
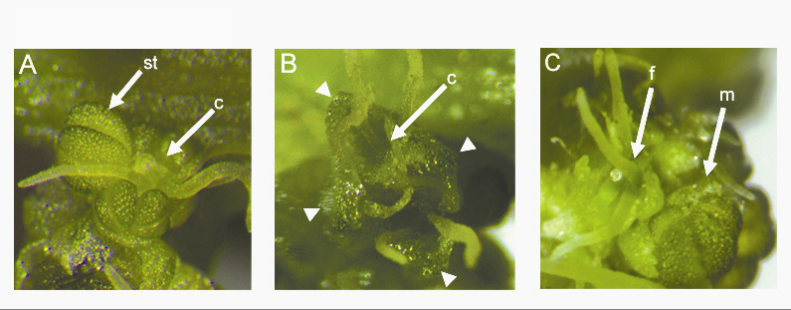
**SpPI-silenced flowers**. a. Mixed flower with stamen (st) and fourth whorl carpel (c). b. Flower with four sepals (indicated by arrowheads) and four carpels in the third whorl. c. Adjacent male (m) and female (f) flowers.

Approximately five to six weeks post inoculation with *pWSRi:SpPI*, all plants had transitioned to flowering, with approximately half developing as male and half developing as female plants. The female *pWSRi:SpPI *plants all developed normal female flowers, and produced seed following pollination. All *pWSRi:SpPI *treated plants that developed into male plants produced flowers with phenotypic defects. Several flowers had active growth in the fourth whorl, some with full carpel development (Figure 2a). Other flowers exhibited homeotic transformations of stamens into carpels in the third floral whorl. As seen with *pWSRi:SpAP3 *male plants, the stamens in the mixed organ flowers sometimes arrested development and did not produce pollen. Several flowers had complete homeotic transformations of stamens to carpels, developing a ring of four carpels with four outer whorl sepals (Figure 2b).

The earliest flowers in *pWSRi:SpPI *males developed mostly male floral organs, whereas flowers produced later tended to show progressively more severe transformations of organ identity. Inflorescences in the upper portion of the plant had a range of flowers, including male flowers, mixed organ flowers, and female flowers. Most flowers at the shoot apex developed as normal females, indicating a complete transformation of floral identity from male to female (Figure 2c). These results indicate that *SpPI *and *SpAP3 *have prominent roles in the regulation of sexual dimorphism beyond homeotic transformation of single organs.

### qRT-PCR and *in situ *characterization of *pWSRi:SpPI *infected plants demonstrate that *SpPI *mRNA levels are specifically decreased

To determine whether the phenotypic results obtained in *pWSRi *treated plants were associated with gene specific knockdown, we quantified the relative amounts of *SpPI *mRNA in inflorescences of male plants treated either with *pWSRi *or *pWSRi:SpPI*. cDNA was prepared from total RNA extracted from inflorescences. Spinach *G6pdh *mRNA was targeted as the control gene to be compared with *SpPI *mRNA levels. The ct values are given in Table 1. The delta-delta ct value was 1.113, which is significant at the p < 0.001 level. This corresponds to a reduction in relative *SpPI *mRNA in the *pWSRi:SpPI *treated plant of approximately 55% compared to the control. This level of knockdown is in the lower range for *pWSRi *gene silencing reported elsewhere [29], but is consistent with the mixture of phenotypically wild type and mutant flowers found in the inflorescence. These results indicate, therefore, that the level of *SpPI *mRNA was specifically reduced in plants treated with *pWSRi:SpPI*.

**Table 1:** qRT-PCR analysis of *pWSRi:SpPI *treated plants.

Treatment	Mean ct *G6pdh*	Mean ct *SpPI*	Δ ct	Δ Δ ct
*pWSRi*	20.565 ± 0.166	17.465 ± 0.105	-3.100	1.113***
*pWSRi:SpPI*	21.192 ± 0.307	19.205 ± 0.118	-1.988	

Mean threshold cycle number ± standard deviations and difference values are listed. *** significant at p < 0.001

**Figure 3:**
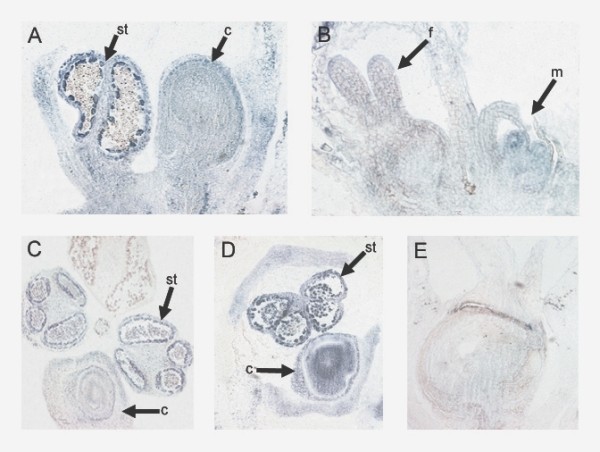
**Plants treated with *pWSRI:SpPI *were fixed, imbedded and sectioned for *in situ *hybridization**. (a, b) Hybridization with antisense RNA SpPI probe. a. Longitudinal section showing third whorl stamen and third whorl carpel. Strong *SpPI *expression is present in tapetal cells in stamen, whereas no detectable staining in the carpel. b. Inflorescence cluster with female (f) and early male flower. *SpPI *is detected in stamen primordium. c. Hybridization with antisense RNA *SpAP3 *probe. Cross section through a single flower with three stamen and one carpel in third whorl. Strong *SpAP3 *expression is detected in developing stamen. *SpAP3 *expression is absent in dehiscing stamen. No discernible *SpAP3 *staining in carpel. d. Hybridization with antisense *SpAG *probe. Cross section of flower with stamen and carpel. Strong *SpAG *expression is detectable in both organs. e. Hybridization with sense RNA *SpPI *probe. Abbreviations: st, stamen; c, carpel; f, female; m, male.

To assess how infection with *pWSRi:SpPI *affected spatial expression in relation to morphological variation, infected plants were prepared for *in situ *hybridization. After plants were scored for phenotypes, *pWSRi:SpPI *male inflorescences were fixed, imbedded and thin sectioned. The sections were hybridized with digoxigenin labeled antisense RNA probes of *SpPI*, *SpAP3*, and *SpAG*. In sections of flowers with mixed organs, *SpPI *was expressed in the stamens, but not in the carpels. Figure 3a shows a longitudinal section through a flower with a stamen and an ovary both in the third whorl. The stamen has pollen grains in the locules. The tapetal cells surrounding the locules are strongly stained, indicating *SpPI *expression. In contrast, there is no *SpPI *hybridization detected in the ovary opposite the stamen. Although occurring in differentiated organs in a single whorl, the *SpPI *expression patterns are similar to those reported in the comparable organs in wild type plants [27]. Figure 3b shows an early stage 5 female flower (marked f) and a late stage 3 male flower (marked m) in the same inflorescence cluster. As in wild type, there is no detectable expression of *SpPI *in the female flower. In contrast, there is *SpPI *hybridization in clusters of L2 cells in the region of the incipient stamen primordium. Hybridization of *SpPI *sense RNA probes to *pWSRi:SpPI *sections gave no signal (Figure 3e). These results demonstrate that suppression of *SpPI *expression correlates with homeotic organ transformation within a single flower, and perhaps induction of complete female flower development on a male plant.

In *Arabidopsis AP3 *and *PI *work as an obligate heterodimer that is required for maintenance of both genes' expression [30]. If one B class gene is not expressed, then the other gene will not be expressed after its initial induction. To test how silencing of *SpPI *affected *SpAP3 *expression, *pWSRi:SpPI *male flowers with mixed organs were hybridized with a probe for *SpAP3*. Figure 3c shows a cross section through a flower with three stamens and a carpel in the third whorl. As with *SpPI*, *SpAP3 *was only expressed in the tapetal cells surrounding the vacuoles in the near-mature anthers. Although also in the third whorl, the ovary does not display any *SpAP3 *expression. Given that this tissue was silenced only for *SpPI*, the expression patterns indicate that *SpPI *is necessary for *SpAP3 *expression as was previously found in *Arabidopsis*.

Both to test for any regulatory interactions between *SpPI *and *SpAG *and to serve as a positive control, *pWSRi:SpPI *mixed flowers were hybridized with antisense RNA probes for *SpAG*. As previously reported in wild type flowers [27], *SpAG *is expressed in both anthers and in the ovary. Figure 3d is a cross-section through a flower with an ovary opposite at stamen. *SpAG *is detected in the developing ovule as well as in the stamen. There is strong expression in a ring of cells surrounding the center of the ovule that likely corresponds to the nucellus. There is weak or background expression in the surrounding integuments and ovary wall. Thus, expression of *SpAG *was unaltered from what we have previously reported. This shows that *SpPI *is not required for regulation of the C class gene in spinach. In concert, the quantitative RT-PCR and *in situ *hybridization results indicate that the phenotypic effects found in *pWSRi:SpPI *treated plants is directly associated with a gene specific knockdown of *SpPI *mRNA.

### There is no evidence of gender-specific allelic states in *SpPI *or *SpAP3*

Previous studies of B class expression [26] and the present results indicate that regulation of B class genes functionally differentiates male and female flower development in spinach. The results do not, however, distinguish between gender-specific trans- or cis regulatory effects, the latter of which could be detected as allelic differences in the *SpPI *and/or *SpAP3 *loci in male versus female individuals. To test for allelic variation, especially in LEAFY binding regions, we isolated genomic DNA from three male and three female individuals for both *SpPI *and *SpAP3 *DNA analysis. A combination of regular and splinkerette [31] PCR was performed to obtain full genomic sequences. After obtaining the complete sequence from a single individual, primers were designed such that sequential regions were amplified so as to overlap with adjacent regions. As a result, all sections of the genes were isolated in at least two independent PCR reactions for each individual surveyed. Sequences were determined from amplified products rather than clones to avoid sampling error or cloning induced artifacts.

**Figure 4:**
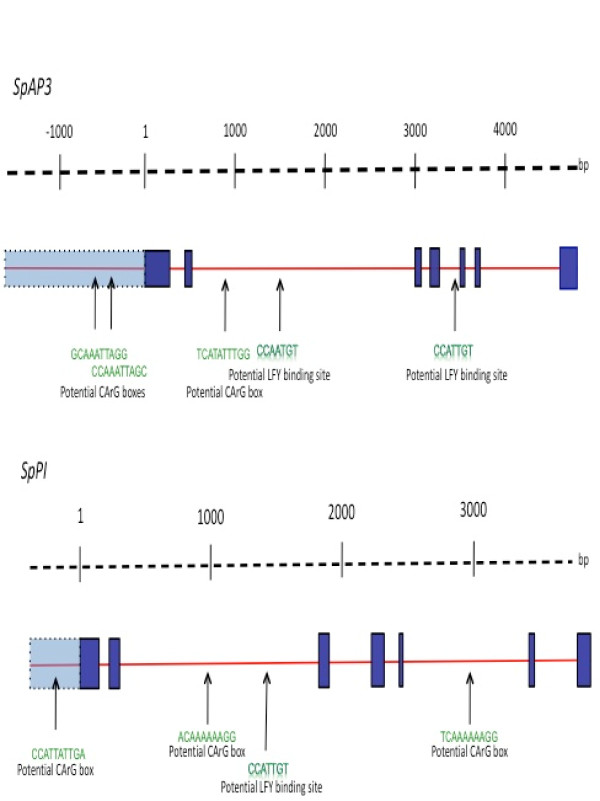
**Schematic of *SpAP3 *and *SpPI *gene structures**. Dark blue boxes represent amino acid encoding regions. Light blue boxes represent 5' UTR and control regions. Thin red lines represent introns. *SpAP3 *has seven exons and six introns. The introns are 153 bp, 2522 bp, 96 bp, 295 bp, 103 bp, and 943 bp in length respectively. Due to the lack of reliable 5' RACE data, the first exon start site is uncertain, however, the coding region of exon 1 extends 191 bp starting from the first position of the start codon. The remaining exons 2, 3, 4, 5, and 6 are 67 bp, 62 bp, 100 bp, 42 bp, and 45 bp in length, respectively, while exon 7 starts 138 bp through the stop codon and continues another 238 bp past the stop codon, although only 182 bp of this 3' untranslated region was included in the present survey. *SpPI *has sevens exons and six introns. The introns are 117 bp, 1504 bp, 388 bp, 127 bp, 912 bp, and 253 bp in length, respectively. Exon 1 extends to position 188 starting from the first position of the start codon. Exons 2 through 6 are 67 bp, 62 bp, 100 bp, 30 bp, and 45 bp long, respectively. Exon 7 starts 120 bp before the end of the stop codon and continues 169 past the stop codon. Positions of potential LEAFY binding elements and CArG boxes are indicated.

The intron-exon structure of the spinach B class genes was predicted based on a comparison with previously published cDNA sequences. We obtained 6676 bp of sequences for *SpAP3 *starting 1737 bp upstream (5') of the start codon through to 182 bp downstream (3') of the stop codon (Figure 4). The gene appears to contain seven exons and six introns. The sequence has been submitted to GenBank under accession number GQ120477.

Using a similar approach, we isolated 4309 bp of the *Sp PI *gene through to the end of the stop codon (Figure 4). We sequenced 396 bp upstream of the start codon. As in *SpAP3, SpPI *has six introns and seven exons. The sequence has been submitted to GenBank under accession number GQ120478.

In both *SpAP3 *and *SpPI*, we did not detect any sequence variation among the six individuals sequenced, including all coding and non-coding regions. To determine if there was any variation that was obscured in a heterozygous state in the direct sequencing, we cloned the 5' non-coding region of *SpAP3*, and sequenced eight individual clones each from one male and one female individual. We anticipated that if the differential regulation of transcription between the sexes were driven by allelic differences, they would be apparent in promoter regions. As with the direct sequencing, all sequences were identical. As the plants used are from a cultivated variety, we anticipated that there may be low sequence variation, however, the complete absence of detected variability even in the large introns and 5' untranslated regions was unanticipated, and reflects the inbred nature of the cultivated variety.

We further scrutinized our sequences to determine whether potential cis regulatory sites could be found (Figure 4). Both LEAFY and MADS box proteins (AP1 and AP3/PI dimers) regulate *AP3 *and *PI *in *Arabidopsis*. Three CArG binding sites have been identified in the 5' region in *Arabidopsis AP3 *[32,33]. In the spinach *AP3 *sequences, there are two potential CArG boxes at sequence positions (relative to the start codon) -708 (GCAAATTAGG) and -384 (CCAAATTGC). A third potential CArG box (TCATATTTGG) is located in the second intron at position 785. Similarly. LEAFY binding sites have been determined in intronic regions in *Arabidopsis *B class genes [34,35]. The spinach *SpAP3 *sequences have one potential LEAFY binding site in the second intron, (CCAATGT) at position 1372, and one in the fourth intron (CCATTGT) at position 3465. In comparison, *SpPI *has three potential CArG boxes: (CCATTATTGA) position -30 in the 5'UTR, (ACAAAAAAGG) position 1083 in the second intron, and (TCAAAAAAGG) position 3052 in intron 5. We detected a single potential LEAFY binding site (CCATTGT) in the second intron at position 1521. Thus, the sequence data indicate the existence of conserved potential cis regulatory elements in both male and female genes.

### SpAG specifies organ identity in the third and fourth whorls, specifies determinacy, and promotes stamen fertility

**Figure 5:**
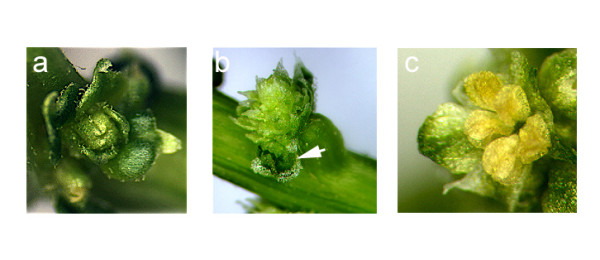
**SpAG-silenced flowers**. a. Flower with complete loss of stamens or carpels in a female silenced plant. b. New inflorescence meristem emerging from center of flower on a male plant. Adjacent flower has four sepals and four opposite sterile organs (arrow). c. Stamens of male flower that failed to mature and produce pollen.

The spinach C class homologue, *SpAG*, is initially expressed throughout the early floral meristem in both males and females. However, as organ primordia begin to develop, *SpAG *takes on a sex-specific expression pattern. Given that *SpAG *is expressed in both developing stamens and carpels and given the apparent lack of regulation by B class genes demonstrated in *pWSRi:SpPI *plants, we wished to determine the functional role of *SpAG *in developing flowers. Spinach plants were treated with *pWSRi:SpAG *coated tungsten particles. Approximately six weeks after infection, plants began to develop phenotypically abnormal flowers. In females, extreme floral abnormalities developed in which floral organs were transformed into bract or leaf-like organs bearing trichomes (Figure 5a). The organs tended to be arranged in a spiral phyllotaxy rather than in distinct whorls. The total number of floral organs increased to well above the normal three found in females, indicating a loss of determinacy in the flower. Several flowers produced continuous whorls of sepals, and then developed an entirely new inflorescence meristem from within the last whorl. Male flowers also often developed a new inflorescence meristem from the center of a flower (Figure 5b). These inflorescences were correctly structured and later produced flowers with *SpAG*-silenced phenotypes. Male *SpAG*-silenced plants also produced flowers that contained modified third whorl organs. The male flower in Figure 5b next to the inflorescence has flat, sterile green organs instead of stamens. Other male flowers developed stamens, however these appeared to have stunted development and never produced pollen (Figure 5c). Therefore, the spinach C class gene has organ identity, microsporangial development, and floral determinacy functions similar to those reported in *Arabidopsis*. However, as anticipated from the *Arabidopsis *model, there were no instances in which flowers switched sex, indicating that regulation of the C class gene is not involved in the sex-determination pathway in this species.

## Discussion

Analysis of sex determination in plants must begin with a clear understanding of where in the developmental process the gender commitment is established. This commitment stage will define what genes are already activated and hence not involved in sex determination, and which ones are yet to be activated, and hence are potential regulation points. *Zea mays*, *Rumex acetosa*, and *Silene latifolia *all begin floral development with both stamens and carpels. In all of these cases, B and C class floral organ identity genes are expressed early in male and female flowers and thus are not involved in triggering sexual differentiation [20,22,36,37]. In contrast, in both *Thalictrum dioicum *[25] and *Spinacia oleracea *[26], in which sexual differentiation occurs at the organ inception stage, B and C class floral organ identity are differentially expressed at floral initiation. Within such systems, it is logical to argue that sex determination can be regulated at the level of expression of the BC floral development genes or immediately upstream in the regulatory pathway. However, this can only be tested through functional analysis of these genes in their native context.

### *SpAGAMOUS *retains floral organ identity and meristem determinacy functions in spinach

A single C class gene has been previously described in *Spinacia oleracea *and has been shown to be expressed early throughout the early floral meristem in both males and females [27]. After sepal initiation, *SpAG *is expressed within the incipient stamen meristems in males and in the center of the floral meristem in females. In maturing male flowers, the expression becomes restricted to the pollen mother cells, whereas in the females the expression is found both in the center of the fourth whorl and at the distal tips of the growing gynoecial girdle. In mature females, *SpAG *is expressed in the nucellus [27].

*SpAG *appears to have three main functions in spinach flower development. First, *SpAG *is required to establish reproductive organ identity. In males, the most extremely affected flowers displayed sterile green third whorl structures in place of stamens (Figure 5B). In females, loss of *SpAG *activity resulted in the loss of carpels. Second, in males in which stamen-like structures did develop, no pollen was produced. These results presumably reflect silencing of *SpAG *at the developmental stage when the expression is restricted to the microsporangium (Figure 5C). Therefore, the spinach C class gene appears to be required for microsporogenesis. These observations conform to reports in *Arabidopsis *that *AGAMOUS *controls microsporogenesis through activation of *SPOROCYTELESS *(*NOZZLE*) [38]. We did not detect female flowers with phenotypically deformed ovaries and so we were not able to determine whether late silencing in females resulted in an analogous loss of megagametophyte development from loss of *SpAG *nucellar expression. Third, the spinach C class gene controls floral determinacy. In extreme female phenotypes, floral organs were replaced by bract-like organs, organized in a continuous spiral phyllotaxy. These flowers had an obvious loss of determinacy, as evidenced by the continual formation of new whorls inside the previous whorl. Additionally, *SpAG *silenced males and females both initiated new inflorescence meristems within developing flowers. These results clearly indicate that the C class organ identity and meristem determinacy functions previously described in *Arabidopsis *are conserved in spinach. The male floral phenotype also suggests that once the fourth whorl is suppressed in males, loss of *SpAG *is not sufficient to generate an indeterminate flower.

The phenotype, in which continuous sterile whorls develop, appears to be remarkably similar to the flower reported in the *Arabidopsis ap2 pi ag *triple mutant [39]. As B class genes are not expressed in spinach female flowers [26], the knockdown of *SpAG *should be comparable to the double *ag pi Arabidopsis *mutant in which multiple whorls of sepals develop due to the expanding A class expression. The more leaf-like organs in the spinach *SpAG *knockdown imply that genes homologous to SEPALLATA or AP1/FUL are not being expressed extensively throughout the flower or that they are not sufficient to define tepal identity. Recent work appears to support this hypothesis as the spinach *AP1 *homologue appears to be expressed only at the initiation of the floral meristem and later in the stamens or carpels, but not in the sepals [40].

### Spinach B class genes define organ identity and are involve in sexual determination and sexual dimorphism

We previously reported on the gender-specific expression patterns of both spinach B class genes. Both genes are expressed in males and, while *SpAP3 *is initially expressed at low levels in females, *SpPI *is not expressed in females at any stage [26]. In plants infected with *pWSRi:SpAP3*, female plants were unaffected. This suggests that the low level of wild type *SpAP3 *expression in female flowers is of no functional significance. The B class proteins in *Arabidopsis *and other species are reported to form heterodimers and to be functional only when both are present [41-43]. Additionally, continued expression of these genes beyond their original initiation by inflorescence identity genes is dependent on the PI/AP3 dimer acting to maintain *PI *and *AP3 *expression [44,45]. Similarly, the lack of phenotypic effect in *pWSRi:SpAP3 *treated plants is consistent with the lack of detectable expression of *SpAP3 *and shows that this gene product is not required for proper female development.

Both *SpPI- *and *SpAP3*-suppressed male plants all form at least some mixed organ flowers, with homeotic transformations of stamens into carpels in the third whorl (Figures 1c, 1d, and 2b). Given these homeotic transformations, it seems that B class genes play a similar role in organ identity determination as has been shown in *A. thaliana *and other model species. In flowers of B-suppressed plants, aberrant organs were all in the third whorl, indicating that the fourth whorl had already been suppressed at the time when organ primordia were initiated.

Some flowers in *pWSRi:SpAP3 *and *pWSRi:SpPI *plants had a fourth whorl carpel, lacked third whorl stamens, but produced four tepals (Figure 1f). Other flowers developed as hermaphrodites with organs in the first, third, and fourth whorls (Figures 1e and 2a). Because wild type male flowers have four tepals, as contrasted with female flowers that have two, the fact that hermaphrodite flowers produced four tepals suggests that male identity had been established earlier in floral ontogeny. Lastly, wild type female flowers were also detected on treated male plants suggesting that earlier B class gene silencing can cause a complete switch in sexual development in the flowers.

As a sex-labile species, spinach is able to modify its sex based on environmental conditions [46]. To achieve sexual plasticity, sex determination is presumably regulated by a system capable of integrating inputs into the regulatory pathway, and able to affect downstream structural gene expression based on those environmental cues. As previously argued, the B class genes are attractive candidates as regulators of sex determination in spinach. It has been shown that B class genes in *Arabidopsis *are direct regulatory targets of gibberellic acid (GA) [47], a hormone capable of causing large male bias when applied to spinach [48,49]. Our results in which wild-type female flowers form on B class gene silenced plants indicate that expression of these B class gene functions as a key regulator of sex determination in spinach.

### A Model for Sex Determination and Sexual Dimorphism in Spinach

Charlesworth and Charlesworth [50] proposed a model for the evolution of dioecious species. The steps involved include the initial evolution of a feminizing mutation that represses the formation of viable male gametes, resulting in a gynodioecious population of plants that are either female or hermaphroditic. The second stage involves the development of a masculinizing factor that represses the gynoecium in hermaphrodites, leading to the development of male flowers. The third stage includes the suppression of recombination of the masculinizing and feminizing factors by chromosomal linkage and inversion. The result is the establishment of sex determining superloci that allow for segregation of male and female determining factors within a dioecious population.

Prior to the present work, the specific genetic elements that control sex-determination in spinach have been unknown. Rosa [51] argued that sex was determined by genetic factors in spinach. In a series of papers, Janick and colleagues demonstrated that a male determining element (Y) existed on chromosome 6 [52-55] and a female determining element (X) existed on chromosome 1 [56]. There is no evidence, however, of reduced recombination or chromosomal evolution leading to distinguishable X and Y chromosomes [57,58]. Alternatively, Chailakhyan [48,59] demonstrated that female plants treated with exogenous gibberellic acid (GA) will produce male flowers, indicating that sex determination can be altered by exogenous applications of the plant hormone. Therefore, the regulation of the genetic factors must be coordinated by elements in the GA regulatory network.

**Figure 6:**
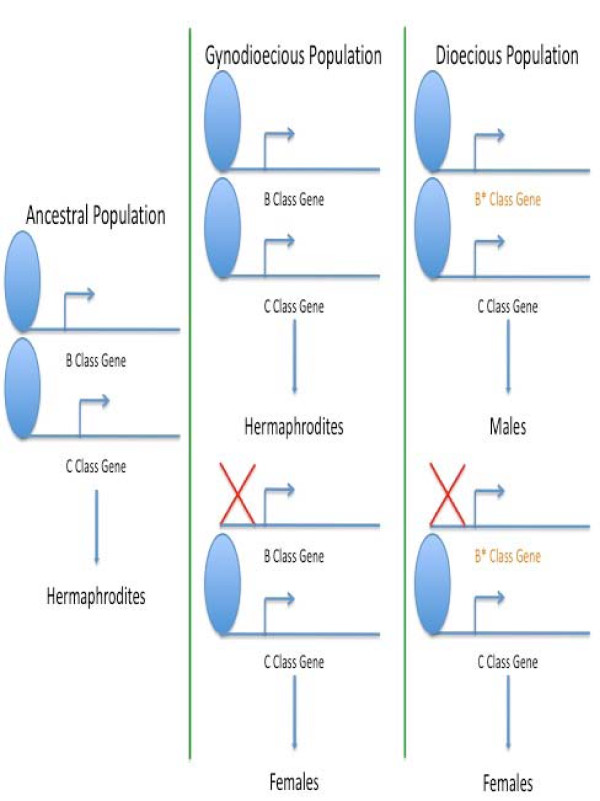
**Model for the evolution of dioecy in spinach**. In the ancestral hermaphroditic species, upstream elements, including but not limited to GA and LFY, activate both B (PI and AP3) and C (AG) class genes. Both classes of genes retain organ identity functions as described in the ABC model. Mutations in the B class genes, notated by *, result in premature termination of the flower in the third whorl, and thus the loss of the carpel. The resultant flower is male. Inactivation or suppression of expression of the B class genes, modulated by the GA response pathway, results in the expression of AG only. The absence of B class gene products causes a reduction in the number of organs in the first whorl and the formation of a single, terminal carpel. The resultant flower is female.

Based on these earlier studies and our present work, we can propose a new model for sex determination in spinach (Figure 6). Pfent et al. [26] demonstrated that the B class genes are only expressed in male flowers and the present study illustrates that suppression of B class expression results in the development of female flowers. This switch of development from male to female flowers is not simply the result of homeosis, as both the number and the whorl location of the organs differ in addition to the organ identity itself. Thus, in spinach, the feminizing mutation must be in the suppression of B class expression. Therefore, an ancestral population in which a gene that regulates the expression of B class genes segregates, would have been gynodioecious, being composed of plants that produced either female (B class genes off) or hermaphroditic (B class genes on) flowers.

We propose that the masculinizing mutation regulated the termination of the flower in the third whorl, rather than in the fourth whorl. Our present study shows that *AG *control of meristem termination is conserved in spinach. Yet, in both spinach and *Arabidopsis*, *AGAMOUS *is expressed in the third whorl. In *Arabidopsis*, this expression does not result in the termination of the flower in the third whorl. However, in spinach, when the B class genes are partially suppressed in male plants, the fourth whorl develops, implying the spinach B class genes are involved in early flower termination and suppression of the fourth whorl. Therefore, the prediction is that the masculinizing mutation, resulting in the suppression of the fourth whorl, occurred in the spinach B class genes or just downstream thereof.

Under this model, there is no requirement for active suppression of recombination. Once the B class genes become fixed in the population, as they appear to be, they will not segregate among individual offspring. The feminizing mutation is then epistatic to the masculinizing mutation. When the B class genes are expressed, male-only flowers develop. When the B class genes are not expressed, female-only flowers develop. Therefore, segregation is only necessary at a single locus. Hence, there is no need or expectation for the evolution of sex chromosomes in spinach.

## Conclusion

We have reported here on the functional characterization of B class floral homeotic genes in a species that develops flowers that are unisexual from inception. While we have identified *SpPI *and *SpAP3 *as a key factors in both floral organ identity and sexual dimorphism in spinach, it is likely that regulation of sex determination originates upstream of the floral organ identity genes. If the regulation of sex determination originates upstream, then the B class genes clearly are key integrating points in the regulatory cascade. Furthermore, it appears that the B-class genes themselves have likely been the loci of the masculinizing mutations that terminate potentially hermaphroditic flowers before they can produce carpels. The evolutionary and developmental mechanisms will become clearer as known regulators of the B class genes are isolated and functionally characterized in spinach and in other species that produce imperfect flowers.

## Methods

### Plant Growth Conditions

Seeds from *Spinacia oleracea *L. cv. America (Twilley Seed Co., Inc., Trevose, PA) were planted in Miracle Gro^© ^potting soil and grown in growth chambers at 20°C under long day conditions (18 h light, 6 hrs dark).

### Construction of *pWSRi:SpAP3*, *pWSRi:SpPI*, and *pWSRi:SpAG*

The *pWSRi *(plasmid Wayne State RNAi) vector was constructed from the Beet Curly Top Virus (BCTV) [29]. The BCTV genome contains two sets of structural genes, termed L and R, which are transcribed from opposite directions toward the center. The multi-cloning site in *pWSRi *containing XhoI and NotI restriction sites was constructed in the center of the genome inside the truncated R3 gene. A 305 base pair XhoI/NotI fragment from the 3' region of *SpPI *was ligated into XhoI/NotI digested *pWSRi *to create the vector *pWSRi:SpPI*. A 263 base pair XhoI/NotI fragment of *SpAP3*, similarly from the 3' region of the gene, was ligated into XhoI/NotI digested *pWSRi *to create the vector *pWSRi:SpAP3*. A 250 bp fragment in the 3' end of *SpAG *was subcloned into pGEM-T-Easy (Promega, Madison, WI, USA), adding XhoI and NotI restriction sites on the ends of the fragment. The XhoI/NotI fragment was then subcloned into XhoI/NotI digested *pWSRi*. Vector clones were verified by sequencing using cycle-sequencing with ABI BigDye^® ^Terminator v3.1 chemistry. The sequencing reaction products were read on an Applied Biosytems ABI Prism 3700 (PE Applied Biosystems, Foster City, CA, USA).

### Biolistic infection of *pWSRi:SpAP3*, *pWSRi:SpPI*, and *pWSRi:SpAG *into spinach plants

Spinach plants were selected at the four-leaf stage before they had transitioned into reproductive growth for biolistic infection of *pWSRi *vectors. Plant were inoculated with *pWSRi *vectors using the Helios™ Gene Gun (Bio-Rad Laboratories, Inc, Hercules, CA, USA). Plasmids were prepared by mixing approximately 10 micrograms of plasmid DNA in sdH_2_O with 20 mg of tungsten powder. The slurry was mixed well, spread on a microscope slide, and the liquid was allowed to evaporate. Bullets were made by first coating plastic tubing (Bio-Rad) with a PVP solution then drying the tubing by a continuous nitrogen gas flush. Plasmid coated tungsten powder was placed in the tubing and allowed to coat the walls. The tubing was then cut to the correct length for use in the Helios™ Gene Gun. Plants were bombarded at 90 PSI in the center of the developing plant from a distance of one inch. Plants were then transferred to a growth chamber at 23°C and grown under short day conditions (8 hours light, 14 hours dark) for three weeks. The plants were then switched to long day conditions and allowed to flower.

### Quantitative Real-time RT-PCR

Total RNA was extracted from male inflorescences of *pWSRi:SpPI *and *pWSRi *(control) treated plants using Trizol following the manufacturer's protocol. Concentration of the total RNA was estimated by spectrophotometry. Total cDNA from approximately one microgram of RNA was made using random hexamers as primers and M-MLV Reverse Transcriptase using standard protocols. One ul RNase H was then added to digest the RNA.

PCR reactions were prepared in batch using the 2× Master Sybr Green PCR mixes (Applied Biosystems) without primers for each cDNA sample so that comparisons between levels mRNA of the control gene (*G6pdh*) and the experimental (*SpPI*) would not be affected by differential cDNA loading errors. Primers were designed to have similar annealing temperatures and to produce products of roughly the same size for each gene. For each cDNA sample, the reaction volumes were then divided into two tubes (4 reactions each), and the specific primer pairs were added. The reactions were then divided into four replicate reactions and placed in staggered wells in the PCR machine. The PCR temperature settings were 94°C for 10 minutes followed by 40 cycles of 94°C 15 seconds, 54°C 15 seconds, and 72°C 45 seconds, and reactions were run and the data collected on a Stratagene Mx3000P. Mean threshold cycles for the *SpPI *and *G6pdh *reactions were calculated for each sample. The delta CT (Threshold cycle_Chelatase _-Threshold cycle_18*S*_) was calculated from the means and the delta CT variances were calculated by summing the individual CT variances as there should be no covariance of the sample errors.

### *In Situ *Hybridization

Plants were harvested approximately six weeks after biolistic infection with *pWSRi *clones and immersed in PROTOCOL 10% Buffered Formalin (Fisher Chemicals, Fairlawn, NJ, USA) at 4°C for ten hours. Inflorescences were dehydrated in a graded ethanol series, cleared in HistoClear^© ^(National Diagnostics, Atlanta, GA, USA), and embedded in Paraplast Plus^© ^(Fisher Chemicals, Fairlawn, NJ, USA) paraffin before sectioning into 8 um sections. Anti-sense and sense strand RNA probes labeled with Digoxigenin-11-UTP were prepared for *SpAP3*, *SpPI*, and *SpAG *as previously described [26,27]. Pre-hybridization clearing, re-hydration, and hybridization were performed according to Ambion's (Austin, TX, USA) mRNA-locator *In situ *Hyb kit. Sectioned inflorescences were hybridized with antisense or sense RNA probes at 50°C for 4 hours. One 2× SSC and two 1× SSC post-hybridization washes of 20 minutes were done at 50°C followed by equilibration in maleic acid buffer (0.1 M maleic acid, 0.15 M NaCl, 0.1% Tween-20, pH 7.5) for ten minutes. Sections were blocked with 0.1% BSA in maleic acid buffer for one hour before a one-hour incubation with a 1:5000 dilution of anti-Digoxigenin antibody conjugated to an alkaline phosphatase (Roche, Indianapolis, IN, USA). Sections were washed four times in maleic acid buffer for twenty minutes before development. Color precipitate was achieved by incubation in a NBT/BCIP solution (Roche, Indianapolis, IN, USA), then stopped by washing in sterile water. Sections were covered in Permount^© ^mounting media (Fisher, Fairlawn, NJ, USA) before the addition of a coverslip. Samples were viewed on a Zeiss compound light microscope with differential interference contrast (Normarski) optics. Photographs were taken on a SPOT RT v3.0 digital camera system and imported into Adobe Photoshop 7.0 for contrast adjustment. Floral stages were described as in Sather, et al., (2005).

### Genomic sequence analysis of B class floral homeotic genes

Genomic DNA from three male and three female individuals was isolated using Promega Wizard Genomic DNA purification kit. To isolate fragments of the *SpPI *gene, nine sets of overlapping primers were designed to search for allelic differences between males and females. For the *SpAP3 *genomic male-female DNA comparison, 15 pairs of overlapping primers were designed and applied to the DNA of three male and three female individuals. Each set of primers was designed to amplify approximately 800 base pairs of genomic DNA with each consecutive primer pair amplifying approximately 400 base pairs of sequence amplified with previous primer set. The sequences of all the primers are listed in Additional file 1: Table S1. PCR reactions were cleaned using Wizard SV Gel and PCR Clean-Up kit, direct sequencing was performed using PI specific primers, and sequences were analyzed using SEQUENCHER program.

## Authors' contributions

DNS executed the gene silencing and *in situ *hybridization experiments, interpreted the data and contributed to the design of the project and to the writing of the manuscript. MJ executed the genetic sequencing and analysis. EMG conceived of the study, contributed to the gene silencing experiments, interpreted the data, and contributed to the writing of the manuscript.

## Supplementary Material

Additional file 1 **Primer pairs used to amplify genomic sequences of the spinach genes *SpPI *and *SpAP3***. This file contains the sequences of the PCR primers used to amplify sequential, overlapping fragments of the genes *SpPI *and *SpAP3*. Click here for file
