# Supplementary material for: Functional analysis of B and C class floral organ genes in spinach demonstrates their role in sexual dimorphism
Source: BMC Plant Biol. 2010 Mar 12;10:46. doi: 10.1186/1471-2229-10-46 (PMC2923521; doi:10.1186/1471-2229-10-46)
Supplement: Additional file 1 — Primer pairs used to amplify genomic sequences of the spinach genes SpPI and SpAP3. This file contains the sequences of the PCR primers used to amplify sequential, overlapping fragments of the genes SpPI and SpAP3. [file 1471-2229-10-46-S1.DOCX]

Supplemental Table 1. Primer pairs used to amplify genomic sequences of the spinach genes *SpPI* and *SpAP3*.

*SpPI* primer pairs

Set 1: ATGTGTCGAACGTGCTCAAG

TTAACTAAACCCTTGTAATATCCTCT

Set 2: CGGTAAGAGGTTGTGGGATG

CGCGCAAGACCTTTTGAA

Set 3: TACAAATGCGATTGCCTTGA

GACCCGGAAAATGAAAGAAA

Set 4: CGCGCACAAGGTGTACAATA

CCCGCATATCGTCATTTTCT

Set 5: CGCCATCCCTAACCCTAACT

CCTCTCCATTCAAGTGCCTA

Set 6: GATATGCGGGTCGAACTCAG

TTTTTGACAACGGCAATAAT

Set 7: AATGACAGGCAGGCAAGTTC

TCGGTTTTTCATACATTTTCACA

Set 8: TATGCTCCGTTCTATTCGACT

ACCAAGACAGAATTGGACAAA

Set 9: GTTTTCGATCTCGACCAAGC

TACACCCTTCCCTGTTGGTT

*SpAP3* primer pairs

Set 1: CAACTGGGCTGCCAACAT

AGAGTGAGCGAGCAAAGAGA

Set 2: AAAAAGCATCAAAACCACACA

GAAGGCGAACGAGCTTAGTG

Set 3: TCCTACGTAGTCCCACACAAAC

TTTGGATCTTGCCTCTCGCCA

Set 4: GTTGCTGTTGAATTTTGATGTTC

GGCCCCAAATATGATCTACTCTT

Set 5: TCCAACAAGCTCCACGAATT

GCCTTTTGGAGGGTGTCTGA

Set 6: TTGGTGTACGGTGCACTCGG

GCTCCTACTCCTTCCCAGTT

Set 7: TCAGACACCCTCCAAAAGGC

CGTTTCTCCAAAACACATCAC

Set 8: AAACTGGGAAGGAGTAGGAGC

TGGTTGTTGCAGTTCCAAAC

Set 9: TTTGCGGTTGTACCAGGGAT

CTTGGGGAATGTGATGCGCGA

Set 10: GTTTGGAACTGCAACAACCA

AATACATGCAAGAAGCAGCTC

Set 11: CAGGGACTTTTACGCCTTGA

CTTGCGTTCACGAATGACCT

Set 12: CTGAAACCCGCCAAAGAGAG

CGGACTGACCTTTTTCTTCG

Set 13: TTCGTGAACGCAAGGTCTAA

CGGTGGGAACAGATTGACTA

Set 14: CCTGCCATTGTGTTGTTGTT

GGGGTTCAAAGGTAACACGA

Set 15: ACGGGGTTCCATCCTAAAAA

TTACACATGATCCTGCACCA
